# The Mitochondrial Unfolded Protein Response: A Hinge Between Healthy and Pathological Aging

**DOI:** 10.3389/fnagi.2020.581849

**Published:** 2020-09-11

**Authors:** Francisco Muñoz-Carvajal, Mario Sanhueza

**Affiliations:** ^1^Center for Integrative Biology, Facultad de Ciencias, Universidad Mayor, Santiago, Chile; ^2^Escuela de Biotecnología, Facultad de Ciencias, Universidad Mayor, Santiago, Chile; ^3^Fondap Geroscience Center for Brain Health and Metabolism, Santiago, Chile

**Keywords:** mitochondrial unfolded protein response, neurodegenerative diseases, aging, mitochondria, epigenetic regulation, stress response

## Abstract

Aging is the time-dependent functional decline that increases the vulnerability to different forms of stress, constituting the major risk factor for the development of neurodegenerative diseases. Dysfunctional mitochondria significantly contribute to aging phenotypes, accumulating particularly in post-mitotic cells, including neurons. To cope with deleterious effects, mitochondria feature different mechanisms for quality control. One such mechanism is the mitochondrial unfolded protein response (UPR^MT^), which corresponds to the transcriptional activation of mitochondrial chaperones, proteases, and antioxidant enzymes to repair defective mitochondria. Transcription of target UPR^MT^ genes is epigenetically regulated by Histone 3-specific methylation. Age-dependency of this regulation could explain a differential UPR^MT^ activity in early developmental stages or aged organisms. At the same time, precise tuning of mitochondrial stress responses is crucial for maintaining neuronal homeostasis. However, compared to other mitochondrial and stress response programs, the role of UPR^MT^ in neurodegenerative disease is barely understood and studies in this topic are just emerging. In this review, we document the reported evidence characterizing the evolutionarily conserved regulation of the UPR^MT^ and summarize the recent advances in understanding the role of the pathway in neurodegenerative diseases and aging.

## UPR^MT^ Machinery and Mitochondrial Homeostasis Regulation

Mitochondria are the main energy producers within the cell and the coordinators of several pathways that control essential metabolites, which include not only ATP and NAD^+^, but also acetyl-CoA and S-adenosyl methionine for protein acetylation and methylation, respectively (Teperino et al., 2010; Menzies et al., 2016). Mitochondria are unique in that they have an independent genome (mtDNA), which encodes 2 rRNAs, 22 tRNAs, and 13 proteins that constitute the OXPHOS complexes (Wallace and Chalkia, 2013). Remaining mitochondrial proteins are encoded in the nucleus, so the function of the organelle heavily depends on the coordinated regulation of nuclear and mitochondrial genomes (Couvillion et al., 2016). Imbalances in protein expression in any of these two sources activate an anterograde regulation of mitochondrial function (from the nucleus towards mitochondria) that adjusts its activity to match cellular needs (Cui et al., 2006; Kaarniranta et al., 2018). Mitochondria can also control the expression of nuclear genes through a retrograde regulatory mechanism (Lin and Haynes, 2016). This bidirectional communication between mitochondria and the nucleus forms a molecular network that maintains cellular homeostasis. Part of the network that synchronizes the cellular adaptation to a variety of stressors is termed the mitochondrial unfolded protein response (UPR^MT^). Thus, UPR^MT^ is the transcriptional program that stabilizes mitochondrial homeostasis and reduces misfolded protein amount in the organelle, increasing the mitochondrial response capability to stress stimuli (reviewed in Jensen and Jasper, 2014; Shpilka and Haynes, 2018; Gomez and Germain, 2019; Tran and Van Aken, 2020). Known activators of UPR^MT^ include the impairment of the Electron Transport Chain (ETC), alteration of mitochondrial dynamics, accumulation of unfolded proteins, deletion of mitochondrial DNA (mtDNA), inhibition of mitochondrial chaperones or proteases, and the increase of reactive oxygen species (ROS) levels (Nargund et al., 2012; Pimenta de Castro et al., 2012; Runkel et al., 2013; Qureshi et al., 2017). Despite the mechanisms underlying the UPR^MT^ are less understood than endoplasmic reticulum UPR (Hetz et al., 2020), this mitochondrial stress pathway is emerging as an important response that guarantees the organelle function.

**Figure 1:**
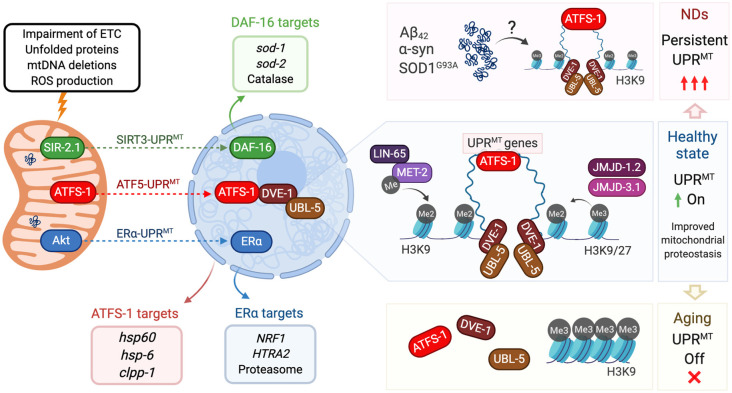
Mitochondrial unfolded protein response (UPR) and its regulation in aging and neurodegeneration. Insults to mitochondria (top left) activate three different axes of the UPR^MT^ program. The SIRT3-UPR^MT^ axis (green arrow) increases the transcription of superoxide dismutases and catalase, after the activation of DAF-16/FOXO3 by the deacetylase SIR-2.1/SIRT3. In the ATF5-UPR^MT^ axis (red arrow), the transcription factor ATFS-1/ATF5 relocalizes from mitochondria to nucleus to upregulate mitochondrial proteostasis related genes (red box) after the interaction with the chromatin stabilizers DVE-1 and UBL-5. In the ERα-UPR^MT^ axis (blue arrow) the estrogen receptor is activated by the kinase Akt, to increase the expression of the protease HTRA2 and mitochondrial biogenesis regulator NRF1. On a healthy state (center right), ATF5-UPR^MT^ activation requires chromatin reorganization. Dimethylation of Histone 3 by MET-2, and presence of demethylases JMJD-1.2 and JMJD-3.1, allow the binding of DVE to facilitate the ATFS-1-dependent expression of UPR^MT^ genes and improve mitochondrial proteostasis. In neurodegenerative states triggered by accumulation of Aβ_42_ (AD), α-syn (PD) or SOD1^G93A^ (ALS, top right), UPR^MT^ could be persistently activated, affecting mitochondria proteostasis and neuron viability. In aging cells (bottom right), Histone 3 is preferentially trimethylated, which blocks DVE and ATFS-1 biding to compacted DNA. Lack of expression of UPR^MT^-related genes decreases mitochondrial response to aging-causing damage. All protein names are taken from *C. elegans*, except the ones from the ERα axis, which has only been described in mammalian cells.

UPR^MT^ was originally observed in mammalian cells, where mitochondrial stress was induced by mtDNA deletions (Martinus et al., 1996) and by aggregation of mutant ornithine transcarboxylase (ΔOTC) (Zhao et al., 2002). Both stress stimuli upregulated the expression of mitochondrial chaperones Hsp60, Hsp10 under the control of the transcription factor CHOP (Zhao et al., 2002; Horibe and Hoogenraad, 2007). Three nuclear components were then identified in *C. elegans* as UPR^MT^ regulators: ATFS-1, DVE-1, and UBL-5. These proteins are part of the UPR^MT^-ATF5 axis, an ATFS-1/ATF5 dependent response that is the most characterized UPR^MT^ pathway (Table 1, Kenny and Germain, 2017; Ji et al., 2020). ATFS-1, a leucine zipper protein, carries a nuclear localization sequence and a mitochondrial targeting sequence. Under mitochondrial stress, ATFS-1 normal transport towards mitochondria is blocked and translocates instead to the nucleus where it interacts with DVE-1 and UBL-5 (Figure 1; Nargund et al., 2012, 2015). In mammals, the CHOP target ATF5 was identified as the functional ortholog for ATFS-1, which also contains targeting sequences for mitochondria and nucleus and upregulates UPR^MT^ genes (Teske et al., 2013; Fiorese et al., 2016). On the other hand, DVE-1 is a DNA binding protein that together with its coregulator UBL-5, interacts with chromatin regions to maintain an ATFS-1-dependent active transcription of UPR^MT^-related genes (Benedetti et al., 2006; Haynes et al., 2007; Tian et al., 2016). The coordinated action of these three proteins upregulates the expression of mitochondrial chaperones hsp-60, hsp-6, and protease clpp-1 (Table 1, Haynes and Ron, 2010).

**Table 1:** Mitochondrial UPR regulators and their function in conserved species.

Name	CE	DM	MM	HS	Function	References
**UPR^MT^-ATF5 axis**	
Activating Transcription Factor 5	Atfs-1	crc	Atf5	ATF5	Transcription factor with basic leucine zipper domain. Carries an MTS in the N-term and an NLS in the C-term.	Yoneda et al. (2004), Nargund et al. (2012), Fiorese et al. (2016) and Wu et al. (2018)
Special AT-Rich Sequence-Binding Protein 2	dve	DVE	Satb2	SATB2	DNA binding protein. Stabilizes open chromatin for UPR^MT^-associated transcription.	Haynes et al. (2007) and Tian et al. (2016)
Ubiquitin Like 5	UBL-5	ubl	Ubl5	UBL5	Protein binding. Binds DVE to activate transcription of Hsp60	Benedetti et al. (2006)
ATP Binding Cassette Subfamily B Member 10	haf-1	CG3156	ABCB10	ABCB10	Mitochondrial inner membrane transporter. Exports peptides from the matrix.	Haynes and Ron (2010) and Yano (2017)
Caseinolytic Mitochondrial Matrix Peptidase Proteolytic	clpp-1	ClpP	ClpP	CLPP	Mitochondrial ATP-dependent protease. Its attenuation reduces the UPR^MT^ activation and the formation of the UBL/DVE complex.	Haynes et al. (2007) and Al-Furoukh et al. (2015)
Translocase of Inner Mitochondrial Membrane 23	timm-23	Tim23	Timm23	TIMM23	Protein transmembrane transporter. Required for full induction of UPR^MT^ mediated by ATFS-1.	Rainbolt et al. (2013)
Lon Peptidase 1	, lonp-1	Lon	Lonp1	LONP1	Mitochondrial protease. Degrades ATFS-1 when imported to mitochondria under stress conditions.	Nargund et al. (2012)
Heat Shock Protein Family D (Hsp60) Member 1	hsp-60	Hsp60A Hsp60B Hsp60C	Hspd1	HSPD1	Mitochondrial heat-shock protease. Upregulated upon mitochondrial stress.	Zhao et al. (2002), Yoneda et al. (2004), Haynes et al. (2007) and Owusu-Ansah et al. (2013)
Heat Shock Protein Family A (Hsp70) Member 9	hsp-6	Hsc70–5	Hspa9	HSPA9	Mitochondrial heat-shock protease. More sensitive to oxidative stress than unfolded protein stress.	Yoneda et al. (2004), Benedetti et al. (2006) and Merkwirth et al. (2016)
**UPR^MT^- ERα axis**	
Estrogen Receptor 1	nhr-107	ERR	Esr1	ESR1	Ligand-activated transcription factor. Regulates the expression of Htra2 and NRF1 after Akt phosphorylation.	Papa and Germain (2011) and Riar et al. (2017)
HtrA Serine Peptidase 2	psmd-9	HtrA2	HtrA2	HTRA2	Serine protease. Protein import checkpoint in IMS. Increased expression upon stress.	Papa and Germain (2011)
Nuclear respiratory factor 1	-	-	Nrf1	NRF1	Transcription factor.	Papa and Germain (2011)
**UPR^MT^- SIRT3 axis**	
Sirtuin 3	Sir-2.1	Sirt2	Sirt3	SIRT3	NAD^+^ dependent deacetylase. Regulates the activity of FOXO3 upon oxidative stress in the mitochondria.	Mouchiroud et al. (2013); Papa and Germain (2014); Gariani et al. (2016) and Kenny et al. (2017)
Forkhead box	daf-16	foxo	Foxo3	FOXO3	Transcription factor. Translocate to the nucleus to activate transcription of SOD1, SOD2, and Catalase.	Mouchiroud et al. (2013); Gariani et al. (2016) and Kenny et al. (2017)
Superoxide dismutase 1	sod-1	Sod	Sod1	SOD1	Superoxide dismutase. Soluble cytoplasmic isoenzyme.	Mouchiroud et al. (2013); Gariani et al. (2016) and Kenny et al. (2017)
Superoxide dismutase 2	sod-2	Sod2	Sod2	SOD2	Superoxide dismutase. Mitochondrial isoenzyme.	Mouchiroud et al. (2013); Gariani et al. (2016) and Kenny et al. (2017)
**UPR^MT^ epigenetic regulators**	
SET Domain Bifurcated Histone Lysine Methyltransferase 1	met-2	eggless	Setdb1	SETDB1	Histone methyltransferase. Loci protected by H3K9 contains genes upregulated upon mitochondrial stress.	Tian et al. (2016)
Abnormal cell lineage.65	lin-65	-	-	-	Nuclear co-factor. Highly unstructured protein involved in chromatin remodeling. Necessary for the incorporation of MET-2 to the nucleus.	Tian et al. (2016)
Euchromatic Histone Lysine Methyltransferase 1	SET-6	CG4565	Ehmt1	EHMT1	Histone methyltransferase. Upregulated during aging to inhibit UPR^MT^ activation.	Yuan et al. (2020)
Lysine Demethylase 6A	JMJD-3.1	Utx	Kdm6A	KDM6A	Histone demethylase. Positive regulator of lifespan upon mitochondrial stress. Needed to activate the UPR^MT^	Merkwirth et al. (2016)
Bromodomain Adjacent to Zinc Finger Domain 2B	Baz-2	tou	Baz2b	BAZ2B	Transcription factor. Neuronal Epigenetic Reader upregulated during aging acting with SAT-6 to inhibit the UPR^MT^ during aging by regulating methylation of H3K9.	Yuan et al. (2020)

*CE, *Caenorhabditis elegans*; DM, *Drosophila melanogaster*; MM, *Mus musculus*; HS, *Homo sapiens*; MTS, mitochondrial targeting sequencing; NLS, nuclear localization signal; IMS, mitochondrial intermembrane space*.

Two other pathways have been associated with this stress response (Figure 1). The UPR^MT^-ERα axis, a pathway dependent on the activation of the estrogen receptor α (ERα), was described as associated with the accumulation of proteins in the mitochondrial intermembrane space (Papa and Germain, 2011). Mitochondrial stress and ROS production trigger the phosphorylation of the protein kinase AKT and consequently, the activation of ERα. This cascade increases the transcription of protease HTRA2 and the mitochondrial biogenesis regulator NFR1, which translates in an increased proteasome activity independent of activation of the UPR^MT^-ATF5 axis (Table 1; Papa and Germain, 2011). Finally, the UPR^MT^-SIRT3 axis is based on the activation of Sirtuin 3 that modulates the expression of SOD1, SOD2, and catalase, through activation of FOXO (Papa and Germain, 2014; Kenny et al., 2017). The UPR^MT^-SIRT3 axis has been validated also in worms and mammalian cells, supporting the high evolutionary conservation of the pathway (Mouchiroud et al., 2013). Importantly, both ERα- and SIRT3-UPR^MT^ axes work independently of CHOP (Papa and Germain, 2014), upholding the idea of three parallel paths coordinating the same stress response (Figure 1).

Chromatin remodeling has been shown to play a central role in UPR^MT^ regulation. Histone 3 is a target for methylation catalyzed specifically by methyltransferase MET-2 in *C. elegans* (ortholog of human SETDB1). Activation of UPR^MT^ requires the dimethylation of lysine 3 of histone 3 (H3K9), which translates into a compacted and overall silenced chromatin state. At the same time although, other chromatin portions remain loose, favoring the binding of UPR^MT^ regulators such as DVE-1 (Tian et al., 2016). Also required for UPR^MT^ activation are the conserved demethylases JMJD-3.1 and JMJD-1.2, which reduce the chromatin compaction by removing methylation from H3K9 and H3K27 (Figure 1; Merkwirth et al., 2016; Sobue et al., 2017). Interestingly, chromatin remodeling acts independently of ATFS-1, as its downregulation does not affect the nuclear localization of DVE-1 (Tian et al., 2016). It is known that besides genes encoding chaperones and proteases, UPR^MT^ activation increases the expression of glycolysis and amino acid catabolism genes, and represses TCA-cycle and OXPHOS encoding genes (Nargund et al., 2015; Gitschlag et al., 2016; Lin and Haynes, 2016). To date, it is not clear whether UPR^MT^ can activate any other quality control mechanism such as mitochondrial fission, fusion, and mitophagy. It has been reported, however, that the same mitochondrial stressors can activate mitophagy and UPR^MT^ (Nargund et al., 2012; Pimenta de Castro et al., 2012; Jin and Youle, 2013; Runkel et al., 2013; Lin et al., 2016). Organisms that have adapted after constant exposure to low doses of these stressors (misregulation of ETC components and low doses of the UPR^MT^ activator paraquat) exhibit a hormetic phenotype as they show increased longevity despite their mild mitochondrial dysfunction (Yoneda et al., 2004; Owusu-Ansah et al., 2013). This homeostatic regulation is particularly important in post-mitotic cells such as neurons.

## The Role and Regulation of UPR^MT^ in Aging

Aging is defined as the time-dependent functional decline that increases vulnerability to different forms of stress, ultimately leading to death (Kennedy et al., 2014). Aging has particularly severe consequences for organs composed mostly by post-mitotic cells, such as the heart and brain (Kowald and Kirkwood, 2000; Terman et al., 2010). For instance, aging is the major risk factor for the onset of chronic, brain-related, and neurodegenerative diseases (ND). Current studies in the field introduced critical questions aiming to understand the physiological sources of time-dependent damage, the compensatory cellular responses that reestablish homeostasis, and their interconnection to find potential targets to intervene and delay aging. Seven cellular pillars of aging have been described, including among others, alterations to proteostasis, epigenetics, metabolism, and adaptation to stress (Kennedy et al., 2014). Mitochondrial dysfunction is a common factor for these events, suggesting a role of mitochondrial reparative machinery in aging progression. Furthermore, it is accepted that aging in model organisms is functionally associated with mitochondrial decline, contributing to the time-dependent tissue malfunction (Chistiakov et al., 2014; Kim et al., 2018). Therefore, activation of UPR^MT^, as one of the mitochondrial mechanisms against different forms of aging-causing damage, could be in part bridging the adaptation to stress and other pillars of aging as proteostasis and epigenetics.

Current evidence highlights an age-dependent effect of UPR^MT^ on lifespan. For instance, activation of UPR^MT^ triggered by downregulation of ETC complexes I and IV promotes longevity (Dillin et al., 2002; Durieux et al., 2011; Mouchiroud et al., 2013). Histone demethylases JMJD-1.2 and JMJD-3.1 mediate in part that extension, as their overexpression is sufficient to extend the lifespan of worms (Merkwirth et al., 2016). On the other hand, reducing the expression of nuclear effectors ATFS-1, UBL-5 and DVE-1, or demethylases JMJD-1.2 and JMJD-3.1, suppresses the lifespan extension (Table 1, Durieux et al., 2011; Houtkooper et al., 2013; Merkwirth et al., 2016; Lan et al., 2019). It is interesting that UPR^MT^ activation after exposure to mitochondrial stress is strongly responsive only during development and not in later stages of the lifespan (Dillin et al., 2002; Copeland et al., 2009; Durieux et al., 2011; Houtkooper et al., 2013). UPR^MT^ appears less active in adult organisms, so there is no increased lifespan as a response to mitochondrial stressors, as observed in developmental stages in worms and flies (Dillin et al., 2002; Owusu-Ansah et al., 2013; Jensen et al., 2017).

Decreased chromatin accessibility of target UPR^MT^ genes in aged organisms is a potential explanation for the differential UPR^MT^ activation. This was recently confirmed in a study where the methyltransferase SET-6 and the neuronal epigenetic reader BAZ-2, mediated specifically an age-dependent regulation of UPR^MT^. Both proteins when overexpressed in aged worms increased the levels of H3K9Me3, the triple methylated state of the protein, thus inhibiting UPR^MT^ activation in the H3K9-protected loci (Figure 1). Loss of function of SET-6 or BAZ-2 increased healthspan but not longevity, a phenotype that was inhibited downregulating UBL-5 or ATFS-1 (Yuan et al., 2020). Histone 3 methylation appears then as a key epigenetic mediator for UPR^MT^ throughout the lifespan (Merkwirth et al., 2016; Tian et al., 2016; Ono et al., 2017). Longitudinal studies have proved that H3K9Me3 increases during aging in mice hippocampus, and inhibition of this methylation state was sufficient to block aging-associated cognitive decline in mice (Snigdha et al., 2016). Advanced knowledge of the loci carrying UPR^MT^ genes on them, will contribute to further understand the lack of UPR^MT^ activation during aging.

## UPR^MT^ in Aging Neurons and Neurodegenerative Diseases

Several factors influence mitochondrial homeostasis in neurons during aging, such as oxidative damage, neuronal localization, and quality control mechanisms. Compared to mitotic cells, neurons are more sensitive to the accumulation of oxidative damage and defective mitochondria (Kowald and Kirkwood, 2000; Terman et al., 2010). Neuronal unique shape, on the other hand, generates a differential mitochondrial distribution required to provide energy at specific compartments (Obashi and Okabe, 2013). Indeed, evidence shows that at nerve terminals, mitochondria are more prone to age-related dysfunction and oxidative damage compared to non-synaptic mitochondria (Lores-Arnaiz et al., 2016; Olesen et al., 2020). Importantly, aging aggravates the difference between these two populations of mitochondria (Borrás et al., 2010; Lores-Arnaiz et al., 2016). The decreased ability of neurons to renew their pool of healthy mitochondria and the lower activity of quality control mechanisms, act synergistically to trigger deleterious consequences in neurons not only in aging but also at earlier stages. In the etiology of the most prevalent ND, shared critical mitochondrial stressors include misfolded and aggregated proteins, impaired mitophagy, and oxidative stress (Niedzielska et al., 2016; Bakula and Scheibye-Knudsen, 2020; Weidling and Swerdlow, 2020). Considering also the number of ND-causative genes associated with mitochondrial dysfunction (Masters et al., 2015; Hardiman et al., 2017; Poewe et al., 2017), quality control mechanisms such as UPR^MT^ emerge as key intervention targets for age-related diseases. However, compared to other mitochondrial response programs (Pellegrino and Haynes, 2015; Pernas and Scorrano, 2016; Misgeld and Schwarz, 2017) or even UPR^ER^ (Hetz et al., 2020), the studies linking UPR^MT^ and NDs are just emerging.

Parkinson’s disease (PD) is caused by decreased dopamine secretion from damaged dopaminergic neurons (reviewed in Poewe et al., 2017). PD pathomechanism is strongly connected to mitochondrial dysfunction and only recently to UPR^MT^ (Franco-Iborra et al., 2018; Chen et al., 2019). Two proteins encoded by PD-causative genes, serine-threonine kinase PINK1 and E3 ubiquitin ligase Parkin, work together to unclutter dysfunctional mitochondria through mitophagy. PINK1 or Parkin downregulation induces decreased mitochondrial respiration and ATP synthesis, degeneration of dopaminergic neurons, and reduced lifespan (Zhu et al., 2013; Moisoi et al., 2014; Tufi et al., 2014; Choi et al., 2015). In *C. elegans*, the downregulation of their orthologs (*pink-1* and* pdr-1*) activates UPR^MT^ as a mitigation mechanism. Without atfs-1 dependent UPR^MT^ activation, lifespan decreases, and dopamine neurons degenerate (Cooper et al., 2017).

PINK1 also interacts with the ERα target HTRA2, mediating its phosphorylation and activation (Plun-Favreau et al., 2007). Interestingly, mutant alleles of HTRA2 were found in PD patients (Strauss et al., 2005; Unal Gulsuner et al., 2014).

PD pathogenesis is strongly connected to the accumulation of α-synuclein (Poewe et al., 2017). αSyn^A53T^ preferentially accumulates in the mitochondria and interacts with the UPR^MT^-regulator ClpP, suppressing its peptidase activity. Overexpression of the protease is sufficient to decrease αSyn^A53T^-associated pathology in mice (Hu et al., 2019). Despite the previous evidence, reports are suggesting a toxic role of UPR^MT^ over-activation. Expression in dopaminergic neurons of an active form of ATFS-1 lacking the mitochondrial target sequence mimics stress conditions with a constant nuclear expression of UPR^MT^ targets. Over-activation of UPR^MT^ shortens lifespan and promotes faulty mitochondria accumulation, a phenotype synergistically increased overexpressing mutant αSyn^A53T^ (Martinez et al., 2017). From the epigenetic point of view, α-synuclein expression in *Drosophila* led to an upregulation of the methyltransferase EHMT2, with an overall H3K9 dimethylation effect (Sugeno et al., 2016). It would be interesting to study whether chromatin remodeling linked to the H3K9Me2 epigenetic mark in this PD model modifies UPR^MT^ activation as previously reported (Merkwirth et al., 2016; Tian et al., 2016).

Amyotrophic lateral sclerosis (ALS) is the most common motor neuron disease and its complex etiology is explained by the almost 30 causative genes that have been linked to familial cases (reviewed in Hardiman et al., 2017). Among these genes, mutations in the superoxide dismutase SOD1 initially unveiled a link between ALS and mitochondrial dysfunction (Rosen et al., 1993). Post-mortem samples of ALS patients show the altered activity of ETC complexes (Bowling et al., 1993), while SOD1 overexpression in transgenic mice causes dysregulated ETC activity, increased ROS production, and diminished mitochondrial Ca^2+^-buffering (Mattiazzi et al., 2002; Brookes et al., 2004). Mutant SOD1^G93A^ localizes in the mitochondrial intermembrane space, which is sufficient to activate two axes of UPR^MT^ *in vivo* (Gomez and Germain, 2019). CHOP is transiently activated in mice’s spinal cord, followed by Akt-dependent phosphorylation of ERα that upregulates NRF1 and proteasome activity (Riar et al., 2017; Gomez and Germain, 2019). This is consistent with recent reports showing that UPR^MT^ activation precedes the onset of ALS and its activity increases throughout disease progression (Pharaoh et al., 2019). Dysregulation of TDP-43, another ALS causative gene, impairs mitochondria in ALS patients, suppresses ETC complex I and activates UPR^MT^ in cellular and animal models. Downregulation of the UPR^MT^ protease LonP1 increased TDP-43 levels, mitochondrial damage and neurodegeneration (Wang et al., 2019). A third ALS-linked mitochondrial protein is CHCHD10, which has an unknown function but its mutant aggregates in mitochondria causing proteotoxic stress, mitochondrial dysfunction and upregulation of the UPR^MT^ regulators CHOP and ATF5 (Anderson et al., 2019). These reports suggest that the accumulation of ALS-associated mutant proteins in mitochondria persistently over activates UPR^MT^, which could be triggering detrimental effects on already stressed neurons (Figure 1).

Alzheimer’s disease (AD) is characterized by key neuropathological hallmarks such as the abnormal accumulation of the amyloid-β (Aβ) peptide (reviewed in Masters et al., 2015). Evidence indicates that oxidative damage and mitochondrial dysfunction have a key role in AD pathogenesis (Butterfield and Halliwell, 2019; Weidling and Swerdlow, 2020), but the relationship between UPR^MT^ and AD has only been recently explored. Aβ accumulation activates UPR^MT^ in human cells and mice (Shen et al., 2020). In *C. elegans*, the sirtuin-activator resveratrol reduced the Aβ-induced toxicity on a Ubl-5 dependent manner, decreasing the amount of Aβ aggregates (Regitz et al., 2016). Further characterization of this finding could provide clues of a potential connection between the two UPR^MT^ axes, and their association to AD. On the other hand, deficiency of the mitochondrial protease PITRM1 induces UPR^MT^, increased Aβ accumulation, and triggered AD-like phenotypes. These were exacerbated by pharmacological inhibition of UPR^MT^ suggesting a protective role of the pathway on Aβ-associated toxicity (Pérez et al., 2020). The expression of UPR^MT^-related genes appear highly increased in post mortem samples of the prefrontal cortex of AD patients (Beck et al., 2016). It would be noteworthy to determine the temporality of this increased expression to understand whether it is an early program persistently activated throughout the disease progression, or a late response triggered by an overall mitochondrial dysfunction. This is especially relevant considering that the expression of the epigenetic regulators of UPR^MT^ EHMT1 and BAZ2B, and therefore inhibition of UPR^MT^, correlates positively with the progression of AD (Zhang et al., 2013; Yuan et al., 2020). Therefore, future studies should try to clarify whether both inhibition and persistent activation of UPR^MT^ contribute to ND pathomechanisms.

## Future Perspectives

Mitochondrial dysfunction is a hallmark of aging and age-related neurodegenerative diseases (Kennedy et al., 2014). As UPR^MT^ activation extends mitochondrial function, further characterization of the pathway will provide stronger hints to understand neuronal homeostasis and healthspan extension. So far, it seems that UPR^MT^ activation is partially modulated by the age-dependent methylation levels of Histone 3. As H3K9 is differentially methylated in specific brain regions (Snigdha et al., 2016), regulation of the UPR^MT^ could differ in distinct neuronal types. This fact raises concerns when thinking about therapeutic approaches since systemic inhibition of UPR^MT^ could be beneficial for cell types with a dysregulated activation of UPR^MT^, but detrimental for another that requires its activation. Therefore, the fine-tuning of UPR^MT^ in different pathogenic contexts will be a crucial consideration for future studies. In the case of PD, AD and ALS, incipient evidence has emerged in the last years highlighting also an over-activation of UPR^MT^ as contributors of the ND pathomechanisms. Future studies on this topic should focus on determining whether known ND causative genes are associated to UPR^MT^ components on an early neurodegenerative stage, or whether UPR^MT^ is only activated on a late, non-reversible stage as a consequence of an overall neuronal decay. Precise pharmacological modulation of the mitochondrial stress response could bring new alternatives to restore compromised neuronal functions, with a prospective increase in the life quality of ND patients and the elderly population.

## Author Contributions

FM-C and MS planned, researched, and wrote the manuscript.

## Conflict of Interest

The authors declare that the research was conducted in the absence of any commercial or financial relationships that could be construed as a potential conflict of interest.

## References

[B1] Al-Furoukh N. Ianni A. Nolte H. Hölper S. Krüger M. Wanrooij S.. (2015). ClpX stimulates the mitochondrial unfolded protein response (UPR^mt^) in mammalian cells. Biochim. Biophys. Acta. 1853, 2580–2591. 10.1016/j.bbamcr.2015.06.016 26142927

[B2] Anderson C. J. Bredvik K. Burstein S. R. Davis C. Meadows S. M. Dash J.. (2019). ALS/FTD mutant CHCHD10 mice reveal a tissue-specific toxic gain-of-function and mitochondrial stress response. Acta Neuropathol. 138, 103–121. 10.1007/s00401-019-01989-y 30877432 PMC6571048

[B3] Bakula D. Scheibye-Knudsen M. (2020). Mitophaging: mitophagy in aging and disease. Front. Cell Dev. Biol. 8:239. 10.3389/fcell.2020.00239 32373609 PMC7179682

[B4] Beck J. Mufson E. Counts S. (2016). Evidence for mitochondrial UPR gene activation in familial and sporadic alzheimer’s disease. Curr. Alzheimer Res. 13, 610–614. 10.2174/1567205013666151221145445 26687188 PMC5977398

[B5] Benedetti C. Haynes C. M. Yang Y. Harding H. P. Ron D. (2006). Ubiquitin-like protein 5 positively regulates chaperone gene expression in the mitochondrial unfolded protein response. Genetics 174, 229–239. 10.1534/genetics.106.061580 16816413 PMC1569816

[B6] Borrás C. Gambini J. López-Grueso R. Pallardó F. V. Viña J. (2010). Direct antioxidant and protective effect of estradiol on isolated mitochondria. Biochim. Biophys. Acta. 1802, 205–211. 10.1016/j.bbadis.2009.09.007 19751829

[B7] Bowling A. C. Schulz J. B. Brown R. H. Jr. Beal M. F. (1993). Superoxide dismutase activity, oxidative damage and mitochondrial energy metabolism in familial and sporadic amyotrophic lateral sclerosis. J. Neurochem. 61, 2322–2325. 10.1111/j.1471-4159.1993.tb07478.x 8245985

[B8] Brookes P. S. Yoon Y. Robotham J. L. Anders M. W. Sheu S.-S. (2004). Calcium, ATP and ROS: a mitochondrial love-hate triangle. Am J. Physiol. Cell Physiol. 287, C817–C833. 10.1152/ajpcell.00139.2004 15355853

[B9] Butterfield D. A. Halliwell B. (2019). Oxidative stress, dysfunctional glucose metabolism and Alzheimer disease. Nat. Rev. Neurosci. 20, 148–160. 10.1038/s41583-019-0132-6 30737462 PMC9382875

[B10] Chen C. Turnbull D. M. Reeve A. K. (2019). Mitochondrial dysfunction in Parkinson’s disease—cause or consequence? Biology 8, 1–26. 10.3390/biology8020038 31083583 PMC6627981

[B11] Chistiakov D. A. Sobenin I. A. Revin V. V. Orekhov A. N. Bobryshev Y. V. (2014). Mitochondrial aging and age-related dysfunction of mitochondria. Biomed. Res. Int. 2014:238463. 10.1155/2014/238463 24818134 PMC4003832

[B12] Choi S. J. Panhelainen A. Schmitz Y. Larsen K. E. Kanter E. Wu M.. (2015). Changes in neuronal dopamine homeostasis following 1-methyl-4-phenylpyridinium (MPP+) exposure. J. Biol. Chem. 290, 6799–6809. 10.1074/jbc.m114.631556 25596531 PMC4358106

[B13] Cooper J. F. Machiela E. Dues D. J. Spielbauer K. K. Senchuk M. M. Van Raamsdonk J. M. (2017). Activation of the mitochondrial unfolded protein response promotes longevity and dopamine neuron survival in Parkinson’s disease models. Sci. Rep. 7:16441. 10.1038/s41598-017-16637-2 29180793 PMC5703891

[B14] Copeland J. M. Cho J. Lo T. Hur J. H. Bahadorani S. Arabyan T.. (2009). Extension of *drosophila* life span by RNA of the mitochondrial respiratory chain. Curr. Biol. 19, 1591–1598. 10.1016/j.cub.2009.08.016 19747824

[B15] Couvillion M. T. Soto I. C. Shipkovenska G. Churchman L. S. (2016). Synchronized mitochondrial and cytosolic translation programs. Nature 533, 499–503. 10.1038/nature18015 27225121 PMC4964289

[B16] Cui L. Jeong H. Borovecki F. Parkhurst C. N. Tanese N. Krainc D. (2006). Transcriptional repression of PGC-1alpha by mutant huntingtin leads to mitochondrial dysfunction and neurodegeneration. Cell 127, 59–69. 10.1016/j.cell.2006.09.015 17018277

[B17] Dillin A. Hsu A. Arantes-oliveira N. Lehrer-graiwer J. Hsin H. Fraser A. G.. (2002). Rates of behavior and aging specified by mitochondrial function during development. Science 298, 2398–2402. 10.1126/science.1077780 12471266

[B18] Durieux J. Wolff S. Dillin A. (2011). The cell-non-autonomous nature of electron transport chain-mediated longevity. Cell 144, 79–91. 10.1016/j.cell.2010.12.016 21215371 PMC3062502

[B19] Fiorese C. J. Schulz A. M. Lin Y. F. Rosin N. Pellegrino M. W. Haynes C. M. (2016). The transcription factor ATF5 mediates a mammalian mitochondrial UPR. Curr. Biol. 26, 2037–2043. 10.1016/j.cub.2016.06.002 27426517 PMC4980197

[B20] Franco-Iborra S. Vila M. Perier C. (2018). Mitochondrial quality control in neurodegenerative diseases: focus on Parkinson’s disease and huntington’s disease. Front. Neurosci. 12:342. 10.3389/fnins.2018.00342 29875626 PMC5974257

[B21] Gariani K. Menzies K. J. Ryu D. Wegner C. J. Wang X. Ropelle E. R.. (2016). Eliciting the mitochondrial unfolded protein response by nicotinamide adenine dinucleotide repletion reverses fatty liver disease in mice. Hepatology 63, 1190–1204. 10.1002/hep.28245 26404765 PMC4805450

[B22] Gitschlag B. L. Kirby C. S. Samuels D. C. Gangula R. D. Mallal S. A. Patel M. R. (2016). Homeostatic responses regulate selfish mitochondrial genome dynamics in *C. elegans*. Cell Metab. 24, 91–103. 10.1016/j.cmet.2016.06.008 27411011 PMC5287496

[B23] Gomez M. Germain D. (2019). Cross talk between SOD1 and the mitochondrial UPR in cancer and neurodegeneration. Mol. Cell Neurosci. 98, 12–18. 10.1016/j.mcn.2019.04.003 31028834 PMC6614005

[B24] Hardiman O. Al-Chalabi A. Chio A. Corr E. M. Logroscino G. Robberecht W.. (2017). Amyotrophic lateral sclerosis. N. Engl. J. Med. 377. 162–172. 10.1056/NEJMra1603471 28700839

[B25] Haynes C. M. Ron D. (2010). The mitochondrial UPR - protecting organelle protein homeostasis. J. Cell Sci. 123, 3849–3855. 10.1242/jcs.075119 21048161

[B26] Haynes C. M. Petrova K. Benedetti C. Yang Y. Ron D. (2007). ClpP mediates activation of a mitochondrial unfolded protein response in *C. elegans*. Dev. Cell 13, 467–480. 10.1016/j.devcel.2007.07.016 17925224

[B27] Hetz C. Zhang K. Kaufman R. J. (2020). Mechanisms, regulation and functions of the unfolded protein response. Nat. Rev. Mol. Cell Biol. 10.1038/s41580-020-0250-z 32457508 PMC8867924

[B28] Horibe T. Hoogenraad N. J. (2007). The chop gene contains an element for the positive regulation of the mitochondrial unfolded protein response. PLoS One 2:e835. 10.1371/journal.pone.0000835 17848986 PMC1950685

[B29] Houtkooper R. H. Mouchiroud L. Ryu D. Moullan N. Katsyuba E. Knott G.. (2013). Mitonuclear protein imbalance as a conserved longevity mechanism. Nature 497, 451–457. 10.1038/nature12188 23698443 PMC3663447

[B30] Hu D. Sun X. Liao X. Zhang X. Zarabi S. Schimmer A.. (2019). Alpha-synuclein suppresses mitochondrial protease ClpP to trigger mitochondrial oxidative damage and neurotoxicity. Acta Neuropathol. 137, 939–960. 10.1007/s00401-019-01993-2 30877431 PMC6531426

[B31] Jensen M. B. Jasper H. (2014). Mitochondrial proteostasis in the control of aging and longevity. Cell Metab. 20, 214–225. 10.1016/j.cmet.2014.05.006 24930971 PMC4274350

[B32] Jensen M. B. Qi Y. Riley R. Rabkina L. Jasper H. (2017). PGAM5 promotes lasting FoxO activation after developmental mitochondrial stress and extends lifespan in *Drosophila*. eLife 6:e26952. 10.7554/eLife.26952 28891792 PMC5614561

[B1000] Ji T. Zhang X. Xin Z. Xu B. Jin Z. Wu J.. (2020). Does perturbation in the mitochondrial protein folding pave the way for neurodegeneration diseases? Ageing Res. Rev. 57, 100–997. 10.1016/j.arr.2019.100997 31816444

[B33] Jin S. M. Youle R. J. (2013). The accumulation of misfolded proteins in the mitochondrial matrix is sensed by PINK1 to induce PARK2/Parkin-mediated mitophagy of polarized mitochondria. Autophagy 9, 1750–1757. 10.4161/auto.26122 24149988 PMC4028334

[B34] Kaarniranta K. Kajdanek J. Morawiec J. Pawlowska E. Blasiak J. (2018). PGC-1α protects RPE cells of the aging retina against oxidative stress-induced degeneration through the regulation of senescence and mitochondrial quality control. The significance for AMD pathogenesis. Int. J. Mol. Sci. 19:2317. 10.3390/ijms19082317 30087287 PMC6121367

[B35] Kennedy B. K. Berger S. L. Brunet A. Campisi J. Cuervo A. M. Epel E. S.. (2014). Geroscience: linking aging to chronic disease. Cell 159, 709–713. 10.1016/j.cell.2014.10.039 25417146 PMC4852871

[B36] Kenny T. C. Germain D. (2017). From discovery of the CHOP axis and targeting ClpP to the identification of additional axes of the UPR^mt^ driven by the estrogen receptor and SIRT3. J. Bioenerg. Biomembr. 49, 297–305. 10.1007/s10863-017-9722-z 28799020

[B37] Kenny T. T. Hart P. Ragazzi M. Sersinghe M. Chipuk J. Sagar M. A. K.. (2017). Selected mitochondrial DNA landscapes activate the SIRT3 axis of the UPR^mt^ to promote metastasis. Oncogene 36, 4393–4404. 10.1038/onc.2017.52 28368421 PMC5542861

[B38] Kim Y. Zheng X. Ansari Z. Bunnell M. C. Herdy J. R. Traxler L.. (2018). Mitochondrial aging defects emerge in directly reprogrammed human neurons due to their metabolic profile. Cell Rep. 23, 2550–2558. 10.1016/j.celrep.2018.04.105 29847787 PMC6478017

[B39] Kowald A. Kirkwood T. B. (2000). Accumulation of defective mitochondria through delayed degradation of damaged organelles and its possible role in the ageing of post-mitotic and dividing cells. J. Theor. Biol. 202, 145–160. 10.1006/jtbi.1999.1046 10640434

[B40] Lan J. Rollins J. A. Zang X. Wu D. Zou L. Wang Z.. (2019). Translational regulation of non-autonomous mitochondrial stress response promotes longevity. Cell Rep. 28, 1050–1062.e6. 10.1016/j.celrep.2019.06.078 31340143 PMC6684276

[B41] Lin Y.-F. Haynes C. M. (2016). Metabolism and the UPR^mt^. Mol. Cell 61, 677–682. 10.1016/j.molcel.2016.02.004 26942672 PMC4779188

[B42] Lin Y.-F. Schulz A. M. Pellegrino M. W. Lu Y. Shaham S. Haynes C. M. (2016). Maintenance and propagation of a deleterious mitochondrial genome by the mitochondrial unfolded protein response. Nature 533, 416–419. 10.1038/nature17989 27135930 PMC4873342

[B43] Lores-Arnaiz S. Lombardi P. Karadayian A. G. Orgambide F. Cicerchia D. Bustamante J. (2016). Brain cortex mitochondrial bioenergetics in synaptosomes and non-synaptic mitochondria during aging. Neurochem. Res. 41, 353–363. 10.1007/s11064-015-1817-5 26818758

[B44] Martinez B. A. Petersen D. A. Gaeta A. L. Stanley S. P. Caldwell G. A. Caldwell K. A. (2017). Dysregulation of the mitochondrial unfolded protein response induces non-apoptotic dopaminergic neurodegeneration in *C. elegans* models of Parkinson’s disease. J. Neurosci. 37, 11085–11100. 10.1523/jneurosci.1294-17.2017 29030433 PMC5688521

[B45] Martinus R. Garth G. Webster T. Cartwright P. Naylor D. Høj P.. (1996). Selective induction of mitochondrial chaperones in response to loss of the mitochondrial genome. Eur. J. Biochem. 240, 98–103. 10.1111/j.1432-1033.1996.0098h.x 8797841

[B46] Masters C. Bateman R. Blennow K. Rowe C. Sperling R. Cummings J. (2015). Alzheimer’s disease. Nat. Rev. Dis. Prim. 1, 1–18. 10.1038/nrdp.2015.56 27188934

[B47] Mattiazzi M. D’Aurelio M. Gajewski C. D. Martushova K. Kiaei M. Flint Beal M.. (2002). Mutated human SOD1 causes dysfunction of oxidative phosphorylation in mitochondria of transgenic mice. J. Biol. Chem. 277, 29626–29633. 10.1074/jbc.m203065200 12050154

[B48] Menzies K. J. Zhang H. Katsyuba E. Auwerx J. (2016). Protein acetylation in metabolism-metabolites and cofactors. Nat. Rev. Endocrinol. 12, 43–60. 10.1038/nrendo.2015.181 26503676

[B49] Merkwirth C. Jovaisaite V. Durieux J. Matilainen O. Jordan S. D. Quiros P. M.. (2016). Two conserved histone demethylases regulate mitochondrial stress-induced longevity. Cell 165, 1209–1223. 10.1016/j.cell.2016.04.012 27133168 PMC4889222

[B50] Misgeld T. Schwarz T. L. (2017). Mitostasis in neurons: maintaining mitochondria in an extended cellular architecture. Neuron 96, 651–666. 10.1016/j.neuron.2017.09.055 29096078 PMC5687842

[B51] Moisoi N. Fedele V. Edwards J. Martins L. M. (2014). Loss of PINK1 enhances neurodegeneration in a mouse model of Parkinson’s disease triggered by mitochondrial stress. Neuropharmacology 77, 350–357. 10.1016/j.neuropharm.2013.10.009 24161480 PMC3878764

[B52] Mouchiroud L. Houtkooper R. H. Moullan N. Katsyuba E. Ryu D. Cantó C.. (2013). The NAD+/sirtuin pathway modulates longevity through activation of mitochondrial UPR and FOXO signaling. Cell 154, 430–441. 10.1016/j.cell.2013.06.016 23870130 PMC3753670

[B53] Nargund A. M. Fiorese C. J. Pellegrino M. W. Deng P. Haynes C. M. (2015). Mitochondrial and nuclear accumulation of the transcription factor ATFS-1 promotes OXPHOS recovery during the UPR^mt^. Mol. Cell 58, 123–133. 10.1016/j.molcel.2015.02.008 25773600 PMC4385436

[B54] Nargund A. M. Pellegrino M. W. Fiorese C. J. Baker B. M. Haynes C. M. (2012). Mitochondrial import efficiency of ATFS-1 regulates mitochondrial UPR activation. Science 337, 587–590. 10.1126/science.1223560 22700657 PMC3518298

[B55] Niedzielska E. Smaga I. Gawlik M. Moniczewski A. Stankowicz P. Pera J.. (2016). Oxidative stress in neurodegenerative diseases. Mol. Neurobiol. 53, 4094–4125. 10.1007/s12035-015-9337-5 26198567 PMC4937091

[B56] Obashi K. Okabe S. (2013). Regulation of mitochondrial dynamics and distribution by synapse position and neuronal activity in the axon. Eur. J. Neurosci. 38, 2350–2363. 10.1111/ejn.12263 23725294

[B57] Olesen M. A. Torres A. K. Jara C. Murphy M. P. Tapia-Rojas C. (2020). Premature synaptic mitochondrial dysfunction in the hippocampus during aging contributes to memory loss. Redox Biol. 34:101558. 10.1016/j.redox.2020.101558 32447261 PMC7248293

[B58] Ono T. Kamimura N. Matsuhashi T. Nagai T. Nishiyama T. Endo J.. (2017). The histone 3 lysine 9 methyltransferase inhibitor chaetocin improves prognosis in a rat model of high salt diet-induced heart failure. Sci. Rep. 7:39752. 10.1038/srep39752 28051130 PMC5209701

[B59] Owusu-Ansah E. Song W. Perrimon N. (2013). Muscle mitohormesis promotes longevity *via* systemic repression of insulin signaling. Cell 155, 699–712. 10.1016/j.cell.2013.09.021 24243023 PMC3856681

[B60] Papa L. Germain D. (2011). Estrogen receptor mediates a distinct mitochondrial unfolded protein response. J. Cell Sci. 124, 1396–1402. 10.1242/jcs.078220 21486948 PMC3078808

[B61] Papa L. Germain D. (2014). SirT3 regulates the mitochondrial unfolded protein response. Mol. Cell Biol. 34, 699–710. 10.1128/MCB.01337-13 24324009 PMC3911493

[B62] Pellegrino M. W. Haynes C. M. (2015). Mitophagy and the mitochondrial unfolded protein response in neurodegeneration and bacterial infection. BMC Biol. 13:22. 10.1186/s12915-015-0129-1 25857750 PMC4384303

[B63] Pérez M. J. Ivanyuk D. Panagiotakopoulou V. Napoli G. D. Brunetti D. Al-Shaana R. (2020). Loss of function of the mitochondrial peptidase PITRM1 induces proteotoxic stress and alzheimer’s disease-like pathology in human cerebral organoids. Mol. Psychiatry. 10.1038/s41380-020-0807-4 32632204 PMC8758476

[B64] Pernas L. Scorrano L. (2016). Mito-morphosis: mitochondrial fusion, fission and cristae remodeling as key mediators of cellular function. Annu. Rev. Physiol. 78, 505–531. 10.1146/annurev-physiol-021115-105011 26667075

[B66] Pharaoh G. Sataranatarajan K. Street K. Hill S. Gregston J. Ahn B.. (2019). Metabolic and stress response changes precede disease onset in the spinal cord of mutant SOD1 ALS mice. Front. Neurosci. 13:487. 10.3389/fnins.2019.00487 31213966 PMC6554287

[B67] Pimenta de Castro I. Costa A. C. Lam D. Tufi R. Fedele V. Moisoi N.. (2012). Genetic analysis of mitochondrial protein misfolding in *Drosophila melanogaster*. Cell Death Differ. 19, 1308–1316. 10.1038/cdd.2012.5 22301916 PMC3392634

[B68] Plun-Favreau H. Klupsch K. Moisoi N. Gandhi S. Kjaer S. Frith D.. (2007). The mitochondrial protease HtrA2 is regulated by Parkinson’s disease-associated kinase PINK1. Nat. Cell Biol. 9, 1243–1252. 10.1038/ncb1644 17906618

[B69] Poewe W. Seppi K. Tanner C. M. Halliday G. M. Brundin P. Volkmann J. (2017). Parkinson disease. Nat. Rev. Dis. Prim. 3, 1–21. 10.1038/nrdp.2017.13 28332488

[B70] Qureshi M. A. Haynes C. M. Pellegrino M. W. (2017). The mitochondrial unfolded protein response: signaling from the powerhouse. J. Biol. Chem. 292, 10500–13506. 10.1074/jbc.r117.791061 28687630 PMC5566509

[B71] Rainbolt T. K. Atanassova N. Genereux J. C. Wiseman R. L. (2013). Stress-regulated translational attenuation adapts mitochondrial protein import through Tim17A degradation. Cell Metab. 18, 908–919. 10.1016/j.cmet.2013.11.006 24315374 PMC3904643

[B72] Regitz C. Fitzenberger E. Mahn F. L. Dußling L. M. Wenzel U. (2016). Resveratrol reduces amyloid-beta (Aβ1–42)-induced paralysis through targeting proteostasis in an alzheimer model of *Caenorhabditis elegans*. Eur. J. Nutr. 55, 741–747. 10.1007/s00394-015-0894-1 25851110

[B73] Riar A. K. Burstein S. R. Palomo G. M. Arreguin A. Manfredi G. Germain D. (2017). Sex specific activation of the ERα axis of the mitochondrial UPR (UPR^mt^) in the G93A-SOD1 mouse model of familial ALS. Hum. Mol. Genet. 26, 1318–1327. 10.1093/hmg/ddx049 28186560 PMC6075578

[B74] Rosen D. R. Siddique T. Patterson D. Figlewicz D. A. Sapp P. Hentati A.. (1993). Mutations in Cu/Zn superoxide dismutase gene are associated with familial amyotrophic lateral sclerosis. Nature 362, 59–62. 10.1038/362059a0 8446170

[B75] Runkel E. D. Liu S. Baumeister R. Schulze E. (2013). Surveillance-activated defenses block the ROS-induced mitochondrial unfolded protein response. PLoS Genet. 9:e1003346. 10.1371/journal.pgen.1003346 23516373 PMC3597513

[B65] Snigdha S. Prieto G. A. Petrosyan A. Loertscher B. M. Dieskau A. P. Overman L. E.. (2016). H3K9me3 inhibition improves memory, promotes spine formation and increases BDNF levels in the aged hippocampus. J. Neurosci. 36, 3611–3622. 10.1523/jneurosci.2693-15.2016 27013689 PMC4804016

[B76] Shen Y. Ding M. Xie Z. Liu X. Yang H. Jin S.. (2020). Activation of mitochondrial unfolded protein response in SHSY5Y expressing APP cells and APP/PS1 mice. Front. Cell Neurosci. 13:568. 10.3389/fncel.2019.00568 31969805 PMC6960128

[B77] Shpilka T. Haynes C. M. (2018). The mitochondrial UPR: mechanisms, physiological functions and implications in ageing. Nat. Rev. Mol. Cell Biol. 10.1038/nrm.2017.110 29165426

[B78] Sobue S. Inoue C. Hori F. Qiao S. Murate T. Ichihara M. (2017). Molecular hydrogen modulates gene expression *via* histone modification and induces the mitochondrial unfolded protein response. Biochem. Biophys. Res. Commun. 493, 318–324. 10.1016/j.bbrc.2017.09.024 28890349

[B79] Strauss K. M. Martins L. M. Plun-Favreau H. Marx F. P. Kautzmann S. Berg D.. (2005). Loss of function mutations in the gene encoding Omi/HtrA2 in Parkinson’s disease. Hum. Mol. Genet. 14, 2099–2111. 10.1093/hmg/ddi215 15961413

[B80] Sugeno N. Jäckel S. Voigt A. Wassouf Z. Schulze-Hentrich J. Kahle P. J. (2016). α-synuclein enhances histone H3 lysine-9 dimethylation and H3K9me2-dependent transcriptional responses. Sci. Rep. 6:36328. 10.1038/srep36328 27808254 PMC5093762

[B81] Teperino R. Schoonjans K. Auwerx J. (2010). Histone methyl transferases and demethylases; can they link metabolism and transcription? Cell Metab. 12, 321–327. 10.1016/j.cmet.2010.09.004 20889125 PMC3642811

[B82] Terman A. Kurz T. Navratil M. Arriaga E. A. Brunk U. T. (2010). Mitochondrial turnover and aging of long-lived postmitotic cells: the mitochondrial-lysosomal axis theory of aging. Antioxid. Redox Signal. 12, 503–535. 10.1089/ars.2009.2598 19650712 PMC2861545

[B83] Teske B. F. Fusakio M. E. Zhou D. Shan J. McClintick J. N. Kilberg M. S.. (2013). CHOP induces activating transcription factor 5 (ATF5) to trigger apoptosis in response to perturbations in protein homeostasis. Mol. Biol. Cell 24, 2477–2490. 10.1091/mbc.e13-01-0067 23761072 PMC3727939

[B84] Tian Y. Garcia G. Bian Q. Steffen K. K. Joe L. Wolff S.. (2016). Mitochondrial stress induces chromatin reorganization to promote longevity and UPR^mt^. Cell 165, 1197–1208. 10.1016/j.cell.2016.04.011 27133166 PMC4889216

[B85] Tran H. C. Van Aken O. (2020). Mitochondrial unfolded protein-related responses across kingdoms: similar problems, different regulators. Mitochondrion 53, 166–177. 10.1016/j.mito.2020.05.009 32502630

[B86] Tufi R. Gandhi S. De Castro I. P. Lehmann S. Angelova P. R. Dinsdale D.. (2014). Enhancing nucleotide metabolism protects against mitochondrial dysfunction and neurodegeneration in a PINK1 model of Parkinson’s disease. Nat. Cell Biol. 16, 157–166. 10.1038/ncb2901 24441527 PMC4199097

[B87] Unal Gulsuner H. Gulsuner S. Mercan F. N. Onat O. E. Walsh T. Shahin H.. (2014). Mitochondrial serine protease HTRA2 p.G399S in a kindred with essential tremor and parkinson disease. Proc. Natl. Acad. Sci. U S A 111, 18285–18290. 10.1073/pnas.1419581111 25422467 PMC4280582

[B88] Wallace D. C. Chalkia D. (2013). Mitochondrial DNA genetics and the heteroplasmy conundrum in evolution and disease. Cold Spring Harb. Perspect Biol. 5:a021220. 10.1101/cshperspect.a021220 24186072 PMC3809581

[B89] Wang P. Deng J. Dong J. Liu J. Bigio E. H. Mesulam M.. (2019). TDP-43 induces mitochondrial damage and activates the mitochondrial unfolded protein response. PLoS Genet. 15:e1007947. 10.1371/journal.pgen.1007947 31100073 PMC6524796

[B90] Weidling I. W. Swerdlow R. H. (2020). Mitochondria in alzheimer’s disease and their potential role in alzheimer’s proteostasis. Exp. Neurol. 330:113321. 10.1016/j.expneurol.2020.113321 32339611 PMC7282957

[B91] Wu Z. Senchuk M. M. Dues D. J. Johnson B. K. Cooper J. F. Lew L.. (2018). Mitochondrial unfolded protein response transcription factor ATFS-1 promotes longevity in a long-lived mitochondrial mutant through activation of stress response pathways. BMC Biol. 16:147. 10.1186/s12915-018-0615-3 30563508 PMC6298126

[B92] Yano M. (2017). ABCB10 depletion reduces unfolded protein response in mitochondria. Biochem. Biophys. Res. Commun. 486, 465–469. 10.1016/j.bbrc.2017.03.063 28315685

[B93] Yoneda T. Benedetti C. Urano F. Clark S. G. Harding H. P. Ron D. (2004). Compartment-specific perturbation of protein handling activates genes encoding mitochondrial chaperones. J. Cell Sci. 117, 4055–4066. 10.1242/jcs.01275 15280428

[B94] Yuan J. Chang S. Yin S. Liu Z. Cheng X. Liu X. (2020). Two conserved epigenetic regulators prevent healthy ageing. Nature 579, 118–122. 10.1038/s41586-020-2037-y 32103178

[B95] Zhang B. Gaiteri C. Bodea L. G. Wang Z. McElwee J. Podtelezhnikov A. A.. (2013). Integrated systems approach identifies genetic nodes and networks in late-onset alzheimer’s disease. Cell 153, 707–720. 10.1016/j.cell.2013.03.030 23622250 PMC3677161

[B96] Zhao Q. Wang J. Levichkin I. V. Stasinopoulos S. Ryan M. T. Hoogenraad N. J. (2002). A mitochondrial specific stress response in mammalian cells. EMBO J. 21, 4411–4419. 10.1093/emboj/cdf445 12198143 PMC126185

[B97] Zhu J.-Y. Vereshchagina N. Sreekumar V. Burbulla L. F. Costa A. C. Daub K. J.. (2013). Knockdown of Hsc70–5/mortalin induces loss of synaptic mitochondria in a *Drosophila* Parkinson’s disease model. PLoS One 8:e83714. 10.1371/journal.pone.0083714 24386261 PMC3875477

